# Total and partial cancer prevalence in the adult French population in 2008

**DOI:** 10.1186/s12885-015-1168-2

**Published:** 2015-03-19

**Authors:** Marc Colonna, Nicolas Mitton, Nadine Bossard, Aurelien Belot, Pascale Grosclaude, the French Network of Cancer Registries (FRANCIM)

**Affiliations:** 1 Isère Cancer Registry, F-38043 Grenoble, France; 2 FRANCIM, F-31073 Toulouse, France; 3 Service de Biostatistique, Hospices Civils de Lyon, F-69003 Lyon, France; 4 Université Lyon 1, F-69100 Lyon, France; 5 Université de Lyon, F-69000 Lyon, France; 6 CNRS UMR 5558, Equipe Biostatistique Santé, F-69310 Pierre-Bénite, France; 7 Institut de Veille Sanitaire, Département des Maladies Chroniques et Traumatismes, F-94410 Saint-Maurice, France; 8 Cancer Research UK Cancer Survival Group, Faculty of Epidemiology and Population Health, London School of Hygiene & Tropical Medicine, London, UK; 9 Tarn Cancer Registry, F-81000 Albi, France

**Keywords:** Partial prevalence, Total prevalence, Cancer burden, Incidence, Survival

## Abstract

**Background:**

To provide estimations of partial and total prevalence of 24 cancer sites in France in 2008. The estimations of partial prevalence were compared with the previous estimations for 2002.

**Methods:**

Nationwide estimations of incidence and survival data from cancer registries were used for partial prevalence. Nationwide incidence and mortality data were used to estimate total prevalence.

**Results:**

At the end of 2008, in France, nearly 3 million people still alive had received a diagnosis of cancer. Of all prevalent cases, 36% were diagnosed 0 to 5 years earlier and 43% diagnosed 6 to 10 years earlier. The cancer sites with the highest prevalence were the prostate, the breast, and the colon-rectum. The changes in partial prevalence over 5 years (2002 to 2008) were considerable (+244,000 cases) and deemed to be highly related to changes in incidence.

**Conclusion:**

The present estimations update the French prevalence data and highlight the burden of cancer in the population, especially in the elderly. The methods of this study had the advantage of using recent incidence and survival data, which is necessary to show sudden changes in incidence trends and changes in survival that impact prevalence.

**Electronic supplementary material:**

The online version of this article (doi:10.1186/s12885-015-1168-2) contains supplementary material, which is available to authorized users.

## Background

Cancer prevalence has been defined as the number of persons alive who had a cancer diagnosis [1]. This prevalence, called total prevalence, concerns a broad and heterogeneous group because it groups people in complete remission (or cured) who are no longer receiving treatment together with people in need of care and surveillance, be it for initial or recurrent cancer. Another indicator, called partial prevalence, is thus needed. It is used for a more accurate determination of prevalent cases that need specialized healthcare [2-5].

The partial prevalence limits the total prevalence to the persons whose diagnosis was made within a given period of time (e.g., within 1, 3, 5, or 10 years). The meaning of this prevalence depends on the cancer site; however, overall, partial prevalence at 1, 3, and 5 years may indicate the number of people in, respectively, initial treatment, clinical surveillance, and complete remission, [4]. Partial prevalence at 10 years indicates mainly, but not exclusively, the number of cured patients.

Although partial prevalence is an essential epidemiological indicator in cancer, the number of related publications remains low [4]. Nevertheless, some global estimates have been recently published [6].

The objective of this article is to provide estimations of total and partial prevalence for 24 cancer sites in the French population at end of 2008 using the most recent incidence, mortality, and survival data.

## Methods

### Partial prevalence

The partial prevalence has been estimated at 1, 3, 5, and 10 years using a combination of incidence and survival values [7]. This prevalence may be written:

$$ {\mathrm{P}}_{\mathrm{x}}\left(\mathrm{n}\right)={\displaystyle {\sum}_{\mathrm{i}=1}^{\mathrm{n}}{\mathrm{I}}_{\mathrm{k}-1}\times {\mathrm{S}}_{\mathrm{k}-1}\left(\mathrm{i}-0.5\right)} $$

where *I*_*x*_ is the annual number of new cases at age *x* and *S*_*x*_*(t)* the survival probability at time *t* after diagnosis of the cases diagnosed at age *x.* The estimations of the National incidence were obtained according to a modeling method by Belot et al. [8]. This method uses the incidence/mortality ratio relative to the area covered by 17 Département-wide registries (nearly 20% of the French population) over the 1975–2008 period (1975–2009 for breast and prostate cancers) together with the National mortality data.

The overall survivals were estimated by year of age on the basis of incident cases diagnosed between 1995 and 2004 and followed-up by 12 registries (nearly 13% of the French population).

The vital status at 10 years (at the closing date of January 1, 2008) could be obtained for 97% of the patients [9]. “All-cancer partial prevalence” was the sum of the partial prevalence values calculated for the 24 cancer sites (Table 1) plus the partial prevalence of all other cancers.

**Table 1:** **Partial cancer prevalence at five years by sex and age of the adult French population**

Sex and site	[15;44]	[45;54]	[55;64]	[65;74]	[75;84]	85 +	15-85+
**Men**							
Lip, oral cavity, pharynx	1118 (9.1)*	5903 (142.7)	9842 (263.1)	4998 (217.4)	2954 (186.2)	656 (140.5)	25470 (104.1)
Esophagus	77 (0.6)	819 (19.8)	2008 (53.7)	1529 (66.5)	1054 (66.4)	169 (36.2)	5655 (23.1)
Stomach	252 (2.1)	747 (18.1)	1568 (41.9)	2152 (93.6)	2094 (132.0)	634 (135.9)	7448 (30.5)
Colon-rectum	1417 (11.6)	4758 (115.0)	14325 (382.9)	19220 (836.1)	19376 (1221.0)	5202 (1114.5)	64297 (262.9)
Liver	161 (1.3)	575 (13.9)	1992 (53.2)	2648 (115.2)	1788 (112.7)	195 (41.7)	7359 (30.1)
Pancreas	139 (1.1)	411 (9.9)	962 (25.7)	1134 (49.3)	768 (48.4)	179 (38.3)	3592 (14.7)
Larynx	258 (2.1)	1827 (44.2)	3862 (103.2)	2757 (119.9)	1664 (104.9)	315 (67.5)	10684 (43.7)
Lung	858 (7.0)	5063 (122.4)	11785 (315.0)	10835 (471.3)	6369 (401.3)	780 (167.2)	35690 (145.9)
Skin melanoma	3125 (25.6)	2765 (66.8)	4032 (107.8)	3750 (163.1)	3268 (206.0)	952 (204.1)	17893 (73.2)
Prostate	104 (0.8)	6119 (147.9)	63906 (1708.3)	105131 (4573.0)	76298 (4808.2)	13801 (2957.1)	265359 (1085.0)
Testis	7252 (59.3)	1638 (39.6)	586 (15.7)	180 (7.8)	72 (4.5)	33 (7.1)	9761 (39.9)
Bladder	229 (1.9)	1572 (38.0)	5899 (157.7)	7842 (341.2)	8446 (532.2)	2356 (504.8)	26344 (107.7)
Kidney	1021 (8.3)	2827 (68.4)	5747 (153.6)	6021 (261.9)	4823 (304.0)	933 (199.9)	21372 (87.4)
Brain, CNS	1224 (10.0)	709 (17.1)	804 (21.5)	465 (20.2)	232 (14.6)	72 (15.4)	3505 (14.3)
Thyroid	2050 (16.8)	1614 (39.0)	2061 (55.1)	1098 (47.7)	479 (30.2)	76 (16.3)	7378 (30.2)
Non-Hodgkin lymphoma	2356 (19.3)	2695 (65.2)	4415 (118.0)	4588 (199.6)	3874 (244.1)	985 (211.0)	18912 (77.3)
Hodgkin disease	2402 (19.6)	673 (16.3)	482 (12.9)	306 (13.3)	201 (12.7)	57 (12.2)	4122 (16.9)
Multiple myeloma	175 (1.4)	683 (16.5)	1881 (50.3)	2438 (106.0)	2396 (151.0)	601 (128.9)	8175 (33.4)
Acute leukemia	911 (7.4)	350 (8.5)	505 (13.5)	365 (15.9)	245 (15.4)	51 (10.9)	2426 (9.9)
CLL	98 (0.8)	546 (13.2)	1829 (48.9)	2429 (105.7)	2355 (148.4)	744 (159.5)	8001 (32.7)
All cancers	28568 (233.6)	45891 (1109.6)	144782 (3870.3)	187083 (8138.0)	146201 (9213.3)	31051 (6653.2)	583576 (2386.2)
**Women**							
Lip, oral cavity, pharynx	563 (4.7)	1634 (37.9)	2628 (67.4)	1695 (64.4)	1457 (60.9)	769 (69.6)	8745 (33.1)
Esophagus	63 (0.5)	181 (4.2)	376 (9.6)	318 (12.1)	411 (17.2)	121 (11.0)	1470 (5.6)
Stomach	203 (1.7)	335 (7.8)	604 (15.5)	913 (34.7)	1366 (57.1)	734 (66.5)	4155 (15.7)
Colon-rectum	1614 (13.3)	4616 (107.1)	10375 (266.0)	13060 (496.4)	17589 (734.8)	9313 (843.3)	56567 (214.0)
Liver	109 (0.9)	177 (4.1)	438 (11.2)	444 (16.9)	510 (21.3)	116 (10.5)	1795 (6.8)
Pancreas	177 (1.5)	332 (7.7)	808 (20.7)	968 (36.8)	976 (40.8)	272 (24.6)	3534 (13.4)
Larynx	102 (0.8)	280 (6.5)	450 (11.5)	353 (13.4)	272 (11.3)	85 (7.7)	1542 (5.8)
Lung	776 (6.4)	2653 (61.5)	4004 (102.7)	2970 (112.9)	2316 (96.7)	428 (38.7)	13147 (49.7)
Skin melanoma	5254 (43.4)	3872 (89.8)	4544 (116.5)	3675 (139.7)	3275 (136.8)	1599 (144.8)	22219 (84.0)
Breast	19808 (163.8)	46379 (1075.6)	56394 (1445.7)	49397 (1878.0)	35032 (1463.5)	12746 (1154.1)	219756 (831.3)
Cervix uteri	4487 (37.1)	3353 (77.8)	1897 (48.6)	1215 (46.2)	1063 (44.4)	360 (32.6)	12374 (46.8)
Corpus uteri	434 (3.6)	2136 (49.5)	6897 (176.8)	7662 (291.2)	6105 (255.0)	1525 (138.1)	24758 (93.7)
Ovary	1218 (10.1)	2296 (53.3)	3539 (90.7)	3056 (116.2)	2208 (92.2)	541 (49.0)	12858 (48.6)
Bladder	76 (0.6)	279 (6.5)	675 (17.3)	1166 (44.3)	1926 (80.5)	1083 (98.1)	5205 (19.7)
Kidney	636 (5.3)	1304 (30.2)	2387 (61.2)	2718 (103.3)	3103 (129.6)	809 (73.3)	10957 (41.4)
Brain, CNS	1059 (8.8)	585 (13.6)	562 (14.4)	385 (14.6)	230 (9.6)	59 (5.3)	2879 (10.9)
Thyroid	7155 (59.2)	5678 (131.7)	6296 (161.4)	3077 (117.0)	1430 (59.7)	159 (14.4)	23795 (90.0)
Non-Hodgkin lymphoma	1490 (12.3)	1941 (45.0)	3423 (87.7)	3850 (146.3)	4053 (169.3)	1250 (113.2)	16006 (60.5)
Hodgkin disease	2544 (21.0)	400 (9.3)	293 (7.5)	194 (7.4)	156 (6.5)	56 (5.0)	3642 (13.8)
Multiple myeloma	132 (1.1)	527 (12.2)	1351 (34.6)	1930 (73.4)	2403 (100.4)	808 (73.1)	7151 (27.0)
Acute leukemia	830 (6.9)	389 (9.0)	435 (11.2)	379 (14.4)	228 (9.5)	68 (6.1)	2329 (8.8)
CLL	63 (0.5)	368 (8.5)	1143 (29.3)	1723 (65.5)	2196 (91.7)	865 (78.3)	6357 (24.0)
All cancers	51897 (429.1)	82884 (1922.2)	115001 (2948.1)	106883 (4063.0)	96079 (4013.7)	37580 (3402.7)	490324 (1854.8)

* Number of cases (proportion per 100 000 persons) - CNS: Central nervous system - CLL: Chronic lymphocytic leukemia.

The determining factors of change in the partial prevalence over 5 years in France between 2002 and 2008 [3] were analyzed using the decomposition method reported by Bashir et al. [10]. This method allows a quantification of the net change taking into account two demographic factors (the population size and aging) in the overall change of the number of prevalent cases. Within the context of prevalence, the net change results from changes in incidence and survival.

### Total prevalence

Total prevalence requires exhaustive survival data on incident cases over a long period of time (or long-term survival data), which is not available in France. Total prevalence was then estimated using the following relationship between prevalence, incidence, and mortality [11]. This relationship relies on the prevalence odds and applies analytical simplifications that assume that deaths from other causes are the same in cancer patients and other persons [See the Appendix in Additional file 1]. The deduced prevalence p(x,u) is thus:

$$ \mathrm{p}\left(\mathrm{x},\mathrm{u}\right)=\frac{\mathrm{CI}\left(\mathrm{x},\mathrm{u}\right)-\mathrm{C}\mathrm{M}\left(\mathrm{x},\mathrm{u}\right)}{1-\mathrm{C}\mathrm{M}\left(\mathrm{x},\mathrm{u}\right)} $$

In this formula *p(x, u)* is the probability for a subject aged *x* born in year *u* of having had a diagnosis of cancer before age *x*; this corresponds to the prevalence estimated at time *t = u + x* providing that the subject is still alive at time *t*. The net risk of cancer *CI(x,u)* and the net risk of death from cancer *CM(x,u)*) correspond, respectively, to the incidence and mortality cumulated between age *0* and age *x* for a cohort born in year *u*. These cumulated risks were estimated from the specific risks using age-cohort Poisson regression models [12] that take into account the effects of age *x* and birth cohort *u* using a smoothing spline [13]. This kind of modeling requires long-term incidence and mortality data. The study considered the National data available since 1975 over the 1975–2008 period; the incidence and mortality rates relative to the period before 1975 were considered zero. The total prevalence estimated herein is therefore a prevalence obtained with 34 years of data. “All-cancer total prevalence” was estimated directly from the incidence and mortality values calculated for the 24 cancer sites plus the values relative to all other cancers.

The estimations of incidence used for partial and total prevalence correspond to all primary invasive cancers. These estimations were obtained by considering the number of incident cases whatever the ranks of the tumors. Thus, site-specific and “all cancer” prevalence is tumor-based not person-based.

## Results

### Partial prevalence

In 2008, the number of prevalent cases diagnosed within the previous five years was greater than 1 million (Table 1). In men, prostate cancer was the most frequent (nearly 285,000 cases). This concerned all age groups except the 15-44-year-olds in whom testis cancer accounted for one quarter of the cases. In women, in all age groups, breast cancer was the most frequent (nearly 222,000 cases). More than half of the prevalent cases of thyroid, central nervous system, testis, and cervical cancers plus acute leukemia and Hodgkin disease occurred before age 55 years. For all cancers in men and women, the 3% threshold was reached circa age 55 years. This percentage exceeded 9% in men aged 75 to 84 years. A percentage close to or greater than 4% was observed for prostate cancer between ages 65 and 84.

**Figure 1:**
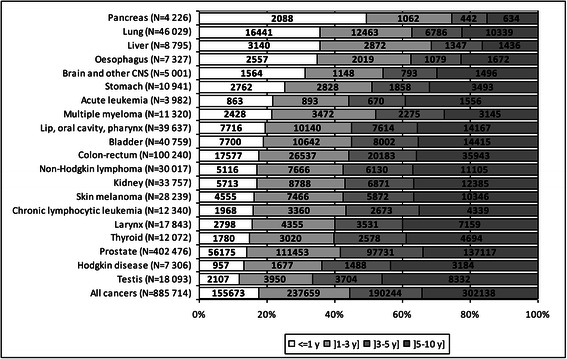
**Breakdown of the prevalent cases at 10 years in men according to the time since diagnosis (France, 2008).**

**Figure 2:**
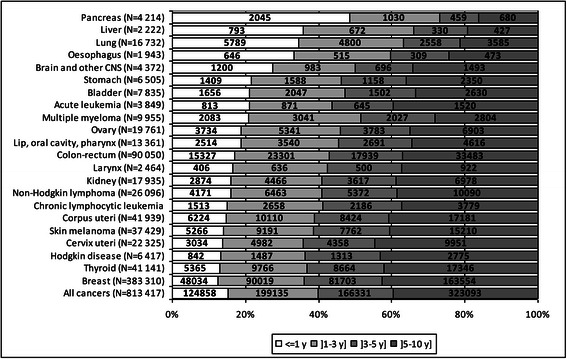
**Breakdown of the prevalent cases at 10 years in women according to the time since diagnosis (France, 2008).**

For all cancers combined, the proportion of cases diagnosed at least 6 years and at most 10 years before was higher in women than in men (Figures 1 and 2). The distribution of the cases according to the time since diagnosis varied with the cancer site. For good prognosis cancers (testis, breast, thyroid in women, and Hodgkin disease), the partial prevalence at 1 year accounted for less than 14% of the partial prevalence at 10 years and the prevalence between 6 and 10 years exceeded 40% of the partial prevalence at ten years. For poor prognosis cancers (pancreas, liver, esophagus, lung), the partial prevalence at 1 year exceeded 30% whereas the partial prevalence between 6 and 10 years ranged between 15 and 25%.

From 2002 to 2008, the5-years partial prevalence of all cancers increased by 37.7% in men and 20.8% in women (i.e., 159,000 and 83,000 cases, respectively; Table 2) which corresponds to a net increase in prevalence of +25.9% in men and +11.3% in women. These increases were mainly due to increases in prostate and breast cancers. The net changes were similar in men and women: decreases of stomach cancers, slight increases in colon-rectum and bladder cancers, and strong increases in liver, pancreas, and thyroid cancers plus melanoma and Hodgkin disease. For lip-mouth-pharynx, lung, esophagus, and larynx cancers, there were decreases in men but increases in women.

**Table 2:** **Breakdown of the five-year prevalence changes (2002 and 2008) according to the main factors: aging and population increase**

	Men						Women					
Cancer site	Cases in 2002	Cases in 2008	Net change (%)	Aging-related change (%)	Population-increase-related change (%)	Overall change (%)	Cases in 2002	Cases in 2008	Net change (%)	Aging-related change (%)	Population-increase-related change (%)	Overall change (%)
Lip, oral cavity, pharynx	27600	25470	−16.7	5.8	3.5	−7.3	6920	8745	15.5	6.0	5.1	26.5
Esophagus	5800	5655	−11.7	6.3	3.8	−1.6	940	1470	43.7	7.9	6.3	58.0
Stomach	7680	7448	−14.4	8.3	3.7	−2.4	4870	4155	−23.9	6.3	3.4	−14.2
Colon-rectum	59280	64297	−3.3	7.8	4.1	8.7	49690	56567	2.8	6.7	4.6	14.1
Liver	4960	7359	39.8	5.4	5.8	50.9	1220	1795	40.2	3.3	6.0	49.4
Pancreas	2370	3592	42.1	5.6	5.9	53.6	2540	3534	30.9	4.6	5.6	41.2
Larynx	10430	10684	−6.9	5.5	3.9	2.6	1150	1542	21.7	6.7	5.3	33.7
Lung	31830	35690	3.0	5.7	4.3	13.0	8100	13147	53.5	3.6	6.5	63.6
Skin melanoma	11750	17893	42.2	4.2	5.8	52.2	16420	22219	26.7	3.2	5.4	35.3
Breast							183780	219756	10.2	4.6	4.8	19.6
Cervix uteri							12600	12374	−6.1	0.6	3.9	−1.6
Corpus uteri							21830	24758	3.3	5.8	4.5	13.6
Ovary							12540	12858	−5.9	4.4	4.1	2.6
Prostate	153380	265359	59.2	7.4	6.6	73.2						
Bladder	23120	26344	1.2	8.8	4.4	14.3	4430	5205	6.5	6.7	4.7	17.9
Kidney	16020	21372	21.7	6.4	5.1	33.2	8570	10957	18.3	4.4	5.1	27.8
Thyroid	4890	7378	42.1	2.5	5.7	50.3	18040	23795	25.0	1.6	5.3	31.8
Non-Hodgkin lymphoma	12050	18912	46.4	5.3	6.0	57.8	11290	16006	32.1	4.3	5.7	42.1
Hodgkin disease	2970	4122	32.6	−1.6	5.2	36.2	2730	3642	30.7	−1.5	5.4	34.6
Multiple myeloma	5640	8175	32.2	7.7	5.6	45.4	5280	7151	24.5	5.9	5.4	35.9
All cancers	424680	583576	25.9	6.5	5.3	37.7	406900	490324	11.3	4.6	4.8	20.8

Testis cancer prevalence was not estimated in 2002 - Acute and chronic lymphocytic leukemias were not distinguished in 2002 - Brain and other CNS cancer definitions were not the same in 2002 and in 2008.

### Total prevalence

The total prevalence amounted to 3 million persons: 1,570,000 men and 1,412,000 women; i.e., respectively, 6.4% and 5.3% of the general French population (Table 3). In men, prostate cancer accounted for one-third of all prevalent cases (2.1% of the adult male population). In women, breast cancer accounted for 46% of all prevalent cases (2.4% of the adult female population). In both men and women, colorectal cancer accounted for nearly 10% of the cases.

**Table 3:** **Total cancer prevalence by sex and age of the adult French population**

Sex and site	[15;44]	[45;54]	[55;64]	[65;74]	[75;84]	85 +	15-85+
**Men**							
Lip, oral cavity, pharynx	2286 (18.7)*	15788 (381.7)	44194 (1181.4)	48011 (2088.5)	44120 (2780.4)	11063 (2370.4)	165462 (676.6)
Esophagus	76 (0.6)	1380 (33.4)	4772 (127.6)	6022 (262.0)	6058 (381.8)	943 (202.1)	19244 (78.7)
Stomach	336 (2.7)	1517 (36.7)	3898 (104.2)	6325 (275.1)	7783 (490.5)	2827 (605.7)	22686 (92.8)
Colon-rectum	2293 (18.8)	8330 (201.4)	27561 (736.8)	45913 (1997.3)	58893 (3711.3)	20558 (4404.9)	163548 (668.7)
Larynx	392 (3.2)	3510 (84.9)	10252 (274.1)	11889 (517.2)	10271 (647.3)	2298 (492.4)	38612 (157.9)
Lung	1180 (9.7)	6343 (153.4)	17011 (454.7)	21498 (935.2)	14615 (921.0)	0 (0.0)	60647 (243.5)
Skin melanoma	5825 (47.6)	6688 (161.7)	9640 (257.7)	8761 (381.1)	7569 (477.0)	2493 (534.2)	40976 (167.5)
Prostate	167 (1.4)	8586 (207.6)	86683 (2317.2)	180306 (7843.5)	185032 (11660.4)	47925 (10268.7)	508699 (2080.0)
Testis	18144 (148.4)	12246 (296.1)	7257 (194.0)	2458 (106.9)	881 (55.5)	95 (20.4)	41081 (168.0)
Bladder	409 (3.3)	2913 (70.4)	12234 (327.0)	21443 (932.8)	30118 (1898.0)	11324 (2426.3)	78441 (320.7)
Kidney	1633 (13.4)	5093 (123.1)	12081 (322.9)	14930 (649.5)	14082 (887.4)	3085 (661.0)	50904 (208.1)
Brain and other CNS	2644 (21.6)	2554 (61.8)	3179 (85.0)	2753 (119.8)	1646 (103.7)	39 (8.4)	12815 (52.4)
Thyroid	4034 (33.0)	4222 (102.1)	5632 (150.6)	3414 (148.5)	1706 (107.5)	228 (48.9)	19236 (78.7)
Non-Hodgkin lymphoma	5906 (48.3)	7687 (185.9)	11912 (318.4)	12496 (543.6)	11225 (707.4)	3069 (657.6)	52295 (213.8)
Hodgkin disease	6418 (52.5)	4742 (114.7)	3739 (100.0)	1640 (71.3)	1060 (66.8)	251 (53.8)	17850 (73.0)
Multiple myeloma	281 (2.3)	1141 (27.6)	3285 (87.8)	4565 (198.6)	5078 (320.0)	1217 (260.8)	15567 (63.7)
CLL	115 (0.9)	842 (20.4)	3379 (90.3)	5546 (241.3)	6599 (415.9)	2137 (457.9)	18618 (76.1)
All cancers	59874 (489.7)	99003 (2393.7)	279135 (7461.8)	450169 (19582.9)	533911 (33646.2)	148788 (31880.2)	1570880 (6423.3)
**Women**							
Lip, oral cavity, pharynx	1219 (10.1)	4150 (96.2)	8128 (208.4)	7170 (272.5)	7252 (303.0)	3912 (354.2)	31831 (120.4)
Esophagus	79 (0.7)	290 (6.7)	764 (19.6)	973 (37.0)	926 (38.7)	259 (23.5)	3291 (12.4)
Stomach	325 (2.7)	734 (17.0)	1566 (40.1)	2320 (88.2)	4163 (173.9)	2331 (211.1)	11439 (43.3)
Colon-rectum	2710 (22.4)	8494 (197.0)	22545 (578.0)	34293 (1303.5)	54171 (2263.0)	32922 (2980.9)	155135 (586.8)
Larynx	162 (1.3)	523 (12.1)	1092 (28.0)	1229 (46.7)	1200 (50.1)	406 (36.8)	4612 (17.4)
Lung	1199 (9.9)	3631 (84.2)	5928 (152.0)	5185 (197.1)	2880 (120.3)	0 (0.0)	18823 (62.7)
Skin melanoma	10791 (89.2)	11171 (259.1)	14507 (371.9)	11986 (455.6)	11008 (459.9)	5144 (465.8)	64607 (244.4)
Breast	28156 (232.8)	90633 (2101.9)	168594 (4322.0)	168779 (6415.5)	137517 (5744.8)	51739 (4684.6)	645418 (2441.5)
Cervix uteri	9051 (74.8)	13615 (315.8)	15171 (388.9)	13428 (510.4)	13129 (548.5)	4887 (442.5)	69281 (262.1)
Corpus uteri	698 (5.8)	3510 (81.4)	14047 (360.1)	24405 (927.7)	28826 (1204.2)	11698 (1059.2)	83184 (314.7)
Ovary	2874 (23.8)	5138 (119.2)	9305 (238.5)	8790 (334.1)	6443 (269.2)	0 (0.0)	32550 (122.0)
Bladder	152 (1.3)	536 (12.4)	1492 (38.2)	2837 (107.8)	5482 (229.0)	3232 (292.6)	13731 (51.9)
Kidney	1184 (9.8)	2501 (58.0)	5157 (132.2)	7030 (267.2)	9620 (401.9)	2822 (255.5)	28314 (107.1)
Brain and other CNS	2512 (20.8)	2251 (52.2)	2574 (66.0)	2334 (88.7)	1719 (71.8)	82 (7.4)	11472 (43.4)
Thyroid	14566 (120.4)	15207 (352.7)	19568 (501.6)	11608 (441.2)	7239 (302.4)	1371 (124.1)	69559 (263.1)
Non-Hodgkin lymphoma	3676 (30.4)	5076 (117.7)	8692 (222.8)	10427 (396.3)	12323 (514.8)	4573 (414.1)	44767 (169.3)
Hodgkin disease	6501 (53.8)	3555 (82.4)	2466 (63.2)	1363 (51.8)	874 (36.5)	318 (28.8)	15077 (57.0)
Multiple myeloma	220 (1.8)	868 (20.1)	2503 (64.2)	3732 (141.9)	4902 (204.8)	1444 (130.7)	13669 (51.7)
CLL	82 (0.7)	578 (13.4)	2160 (55.4)	3884 (147.6)	5980 (249.8)	2667 (241.5)	15351 (58.1)
All cancers	93760 (775.3)	181142 (4201.0)	323253 (8286.8)	347359 (13203.5)	348636 (14564.4)	118133 (10696.1)	1412283 (5342.3)

*Number of cases (proportion per 100 000 persons) - CNS: Central nervous system - CLL: Chronic lymphocytic leukemia.

The distribution of the cases according to the cancer sites varied with age. In men, testis cancer was the most frequent between ages 15 and 44 and lip-mouth-pharynx cancer the most frequent between ages 45 and 54. After age 55, prostate cancer was the most frequent: it concerned more than 10% of the general population aged ≥75 years. In women, breast cancer was the most frequent in all age groups. The prevalence of breast cancer exceeded 5% of the general population aged 65 to 84 years. The second leading cancers were thyroid cancer in the 15–54 age group and colorectal cancer after aged 55; the latter affected 3% of the population aged ≥85 years.

## Discussion

Cancer is a high-burden disease because it affects a high number of individuals and has social and economic consequences. This life-threatening or long-duration disease (depending on the site) requires regular evaluation and anticipation of future needs, especially in terms of healthcare and surveillance.

In France, in 2008, the number of persons with cancer or having had a cancer was close to 3 million, which is nearly 5% of the adult population. The diagnosis of more than one-third of the prevalent cases was made 5 years before and that of 40% made 6 to 10 years before. The cancers with the highest partial and total prevalence were prostate, breast, and colon-rectum cancers. The total prevalence was high in the elderly: more than 30% of men and 11% of women aged ≥ 85 have had cancer.

The partial prevalence was estimated using a classical approach; i.e., annual estimations of incidence by age and observed survival probabilities [6]. Here, this approach had the advantage of using nationwide incidence estimations that are considered valid [14] but it assumed that the incidence/mortality ratio of the registry area is representative of the National ratio. The modeling made it also possible to take into account recent changes in trends [15], especially the stabilization of breast cancer incidence and the marked decrease in prostate cancer incidence since 2004–2005. The survival rate stemming from the registries was also assumed to be a proxy for the National rate. Indeed, the survival data stemmed from 12 Départements (13% of the French population), which is deemed to be acceptably representative.

The analyses of the case distributions according to the time since diagnosis and to the change in prevalence over time are complex because they depend on the initial levels and on the changes in incidence and overall survival, the latter depending itself on cancer-related and other death causes.

Although the prognosis influences the distribution of cases according to the time since diagnosis –a good prognosis led to a high number of prevalent cases beyond 5 years– there are nevertheless particular circumstances that may be explained by changes in incidence. For example, larynx cancer in men, that had a much lower survival rate than skin melanoma [9], had also a higher prevalence beyond 5 years. This can be explained by the inverse trends in incidence of these two cancers: a decrease in larynx cancer vs. an increase in melanoma [15] in a context of stable survival [16]; this translated into higher recent incidence cases of melanoma vs. larynx cancer. The age at incidence influences also the distribution of prevalence according to the time since diagnosis. For example, though it has a good prognosis, prostate cancer presents a distribution that is not favorable because patients are diagnosed late in life, which results in a high number of deaths from other causes than cancer.

The changes in prevalence observed between 2002 and 2008 can be explained by changes in the incidence of some cancers. Indeed, the decrease or stabilization in the incidence of alcohol- and tobacco-related tumors (lip-mouth-pharynx, lung, esophagus, and larynx cancers) in men and their increase in women [15] may explain the sex-linked changes in the prevalence of these cancers. The increase in the incidence of skin melanoma, in which the effect of early diagnosis cannot be distinguished from that of an increase in exposure [17], resulted also in an increase in prevalence. The prevalence of thyroid cancer was also on the rise, especially in women. This may be explained by the sharp increase in the incidence of this cancer, the greatest proportion of which results from the diagnosis of good-prognosis tumors in relatively young subjects [18].

The increase in cancer prevalence in France between 2002 and 2008 is largely attributable to changes in the numbers of breast and prostate cancers. The change in the prevalence of these two cancers can be explained by an increase, up to a recent period, in incidence [15] accompanied by improved survivals [9]. As in thyroid cancer, this increase concerns cases with very good prognoses. More generally, in a context of increase in incidence, De Angelis et al. [19] have shown that this increase may be mainly explained by a change in incidence. When incidence plateaus or decreases, changes in survival are the main explanations for changes in prevalence.

Recent changes in cancer incidence and mortality may explain the differences between the present partial prevalence estimations and those provided by Bray et al. [6], these differences being of various magnitudes according to the cancer site. The present study is based on observed incidence data from cancer registries up to 2008 and on cancers diagnosed between 1995 and 2004 with follow-ups until 2007 whereas, in the study of Bray et al., the incidence data did not extend beyond 2003 and the cancers were diagnosed between 1994 and 1999 with follow-ups until 2003. Besides, the longer surveillance period allowed here estimations of partial prevalence at ten years.

The present estimations of the total prevalence used the net risks of incidence and death per cancer, which assumes the same frequency of death from other causes than cancer in diseased and non-diseased populations [11]. A comparison with the Nordic observations [20] and the US estimations [21], based on the “partial prevalence/total prevalence” ratio (partial prevalence at 5 and 10 years; results not shown), found close values of that ratio for most cancer sites. The most marked differences in the “partial prevalence/total prevalence” ratios may have various causes. These ratios are indeed particularly low in France for lip-mouth-pharynx and esophagus cancers which are particularly impacted by the onset of a second cancer [22-24]. Therefore, the risk of cancer may not necessarily match the death from cancer because death may be attributable to the second but not the first cancer. The ways incidence changes in different countries may also explain the differences in “partial prevalence/total prevalence” ratios; e.g.; the incidence of lip-mouth-pharynx and esophagus cancers decreased in France but increased in the Nordic countries and the United States. The reduction of the input in prevalence induces mechanically an increase in the proportion of prevalent cases with old diagnosis. Although the present estimations regarding these cancers may be biased by the potential presence of multiple cancers, it is difficult to quantify this bias.

Total prevalence was not estimated for liver cancer, pancreas cancer, and acute leukemia because the net risk of death from each of these cancers was higher than that of incidence. In fact, a death/incidence ratio > 1 is associated with a poor quality of mortality data and, sometimes, incidence data. Regarding liver cancer, an overestimation of mortality is possible because a number of secondary cancers may have been considered, at death, as primary cancers [8]. An overestimation may also occur when deaths from jaundice are registered as deaths from liver or pancreas cancer. The data relative to death from pancreas cancer might have been unreliable too [25]. Similarly, a lack of specificity regarding death registration with cause “acute leukemia” may lead to an overestimation of death from leukemia. Besides, an overestimation of mortality may combine with underestimation of incidence. This may occur with liver and pancreas cancer because of the lack of clinical arguments to confirm the diagnoses of cancer [26].

There were three cancer sites for which the net risk of incidence was lower than the net risk of death from cancer in persons aged 85 years and more: lung cancer, ovarian cancer, and central nervous system cancer. This discordance between incidence and mortality may receive two explanations. The first is mechanical. Actually, there is a sharp decrease in incidence after 80 years together with a persistent increase in mortality; this leads to net risks of death higher than incidence. This occurs in a context where the prevalence relative to these three cancers is poorly fuelled by new cases because of the bad prognoses of these cancers at such old ages. The second is epidemiological. Indeed, part of the deaths attributed to lung cancer may, in fact, be due to lung metastases derived from past primary non-pulmonary cancers. In elderly people, some lung cancers are not registered as incident cancers because of insufficient clinical investigations whereas these cases may contribute to the number of deaths from lung cancer. Similarly, in elderly women, some ovarian cancers are not fully investigated, which biases their incidence [26]. Finally, the incidence of central nervous system cancers corresponds to invasive types whereas the benign types may also contribute to the number of deaths; this may induce discordance between the net risk of incidence and mortality, a discordance that may aggravate at advanced ages.

The data on incidence and mortality used here for estimating total prevalence cover the whole 1975–2008 period; that is 34 years. This period provides a sufficient hindsight on the majority of cancer sites for which the median age at diagnosis is over 60 years. However, this period may not be sufficient for cancers of good prognosis that occur mainly in young adults such as testis cancer or Hodgkin disease. The total prevalence for these two cancers is thus probably underestimated.

As already mentioned, uncertainties about the total prevalence of some cancer are linked to poor quality of incidence or mortality data registration. Thus, in case of incidence underestimation (liver or pancreas cancer), the partial prevalence may be biased and should be interpreted with caution too because it relies on incidence and survival.

In addition, in principle, an interval estimation of prevalence seems necessary. Unfortunately, such estimation requires the knowledge of observed prevalence and some approximations [27]. The methodology used here for total or partial prevalence may be further refined by future works.

The part of a partial prevalence within the total prevalence gives an indication of the proportion of patients who continue to require care and surveillance (prevalence at 5 years, nearly one third of prevalent cases), the proportion of those who require less care or surveillance, and even those who are cured (prevalence past 10 years, nearly 40% of cases).

## Conclusion

Prevalence is a key epidemiologic indicator that quantifies the importance of the group of patients who have had cancer. In France, in 2008, more the 3 millions were concerned. The distribution of cancer cases according to the time since diagnosis provides an evaluation of the needs in terms of treatment and surveillance among prevalent cases. Furthermore, prevalent cases may have difficulties due to the social and economic consequences of cancer. The present estimations update the French prevalence data. It demonstrated the importance of using the most updated data (incidence and survival) in the estimation process. Finally, such estimations should be regularly made because of changes in the trends of incidence and improvements of survival.

## Additional file

Additional file 1:
**Analytical formulation for the estimation of total prevalence.**
