# Supplementary material for: Total and partial cancer prevalence in the adult French population in 2008
Source: BMC Cancer. 2015 Mar 19;15:153. doi: 10.1186/s12885-015-1168-2 (PMC4369073; doi:10.1186/s12885-015-1168-2)
Supplement: Additional file 1: — Analytical formulation for the estimation of total prevalence. [file 12885_2015_1168_MOESM1_ESM.doc]

**Appendix: Analytical formulation for the estimation of total prevalence**

Within the context of a given cohort, let

*T_I_* be the time of cancer occurrence (incidence)

*T_K_* be the time of death from cancer occurrence (thus, always *T_K_* ≥ *T_I_*)

*T_P_* be the time of death from other cause occurrence (thus, always *T_I_* ≥ *T_P_* in non-cancer persons)

*T = Min (T_K_,T_P_)* be the time of death occurrence.

Supposing:

- Identical distributions of *T_P_* in cancer and non-cancer patients (implicit condition)
- *T_P_* independent from *T_I_* and *T_K_*

And *p(x)* the prevalence at age *x*: $p(x)=P\left(T_{I}\leq x\left|T>x\right.\right)$.

The ratio of *p(x)* to *1-p(x)* may be written:

$$\begin{matrix} \frac{p(x)}{1-p(x)}=\frac{P\left(T_{I}\leq x\left|T\geq x\right.\right)}{P\left(T_{I}>x\left|T\geq x\right.\right)}=\frac{P\left(T_{I}\leq x,T\geq x\right).P\left(T\geq x\right)}{P\left(T_{I}>x,T\geq x\right).P\left(T\geq x\right)} \\ \text{ }=\frac{P\left(T_{I}\leq x,T_{K}\geq x,T_{P}\geq x\right)}{P\left(T_{I}>x,T_{K}\geq x,T_{P}\geq x\right)} \\ \text{ }=\frac{P\left(T_{I}\leq x,T_{K}\geq x\left|T_{P}\geq x\right.\right).P\left(T_{P}\geq x\right)}{P\left(T_{I}>x,T_{K}\geq x\left|T_{P}\geq x\right.\right)P\left(T_{P}\geq x\right)}\text{ } \\ \text{ }=\frac{P\left(T_{I}\leq x,T_{K}\geq x\left|T_{P}\geq x\right.\right)}{P\left(T_{I}>x,T_{K}\geq x\left|T_{P}\geq x\right.\right)} \\ \text{ }=\frac{P\left(T_{I}\leq x,T_{K}\geq x\right)}{P\left(T_{I}>x,T_{K}\geq x\right)}\text{ }(i) \\ \text{ }=\frac{P\left(T_{I}\leq x,T_{K}\geq x\right)}{P\left(T_{I}>x\right)}\text{ }(ii) \\ \text{ }=\frac{P\left(T_{I}\leq x\right)-P\left(T_{I}\leq x,T_{K}\leq x\right)}{P\left(T_{I}>x\right)}\text{ }(iii) \\ \text{ }=\frac{P\left(T_{I}\leq x\right)-P\left(T_{K}\leq x\right)}{P\left(T_{I}>x\right)}\text{ }(iv) \\ \text{ }=\frac{CI(x)-CM(x)}{1-CM_{I}(x)}\text{ , with }CI(x)=P\left(T_{I}\leq x\right)\text{ and }CM(x)=P\left(T_{K}\leq x\right) \end{matrix}$$

(i) because *T_P_* is independant from *T*_I_ et *T_K_*

(ii) because {T_I_>x} is included in {T_K_>x}

(iii) because P(A∩B) = P(A) – P(A∩B^C^)

(iv) because {T_K_≤x} is included in {T_I_≤x }

Thus, $p(x)=\frac{P\left(T_{I}\leq x\right)-P\left(T_{K}\leq x\right)}{1-P\left(T_{K}\leq x\right)}=\frac{CI(x)-CM(x)}{1-CM(x)}$
